# Challenges and dilemmas on universal coverage for non-communicable diseases in middle-income countries: evidence and lessons from Mexico

**DOI:** 10.1186/s12992-018-0404-3

**Published:** 2018-08-24

**Authors:** Armando Arredondo, Alejandra Azar, Ana Lucia Recaman

**Affiliations:** 1 National Institute of Public Health-Mexico, Av Universidad 655, Col., Sta Maria Ahuacatitlan, CP 62508 Cuernavaca, Mexico; 2 La Salle University, Cuernavaca, Mexico

**Keywords:** Normative universal coverage, Effective universal coverage, Challenges, Dilemma, Non-communicable diseases, Middle-income countries

## Abstract

**Background:**

Despite more than 20 years of reform projects in health systems, the universal coverage strategy has not reached the expected results in most middle-income countries (MICs). Using evidence from the Mexican case on diabetes and hypertension as tracers of non-communicable diseases, the effective coverage rate barely surpasses half of the expected goals necessary to meet the challenges that these two diseases represent at the population level. Prevalence and incidence rates do not diminish either; they even grow. In terms of the economic burden, this means that lack of financial protection and catastrophic expense rates have increased, contrary to what could have been expected.

**Discussion:**

As any complex system, health systems present challenges and dilemmas that are difficult to solve. In terms of universal coverage, when contrasting normative coverage versus effective coverage, the epidemiological, cultural, organizational and economic challenges and barriers become evident. Such challenges have not allowed a greater effectiveness of the contributions of state of the art medicine in the resolution of health problems, particularly in relation to diabetes and hypertension.

**Conclusions:**

Despite of the existence of many universal coverage projects, strategies and programs implemented in MICs, challenges remain and, far from disappearing, unresolved problems are still present, even with increasing trends. The model of care based on a curative biomedical approach was enough to respond to the health needs of the last century, but is no longer adapted to the needs of the present century. The dilemmas of continuity vs. rupture require to review and discuss the background and structure of health systems and their underlying models of care. These two elements have not allowed the different coverage schemes to guarantee greater effectiveness in the application of state of the art medicine, nor a greater health care financial protection for patients and their families. We thus can either accept the fragmented health systems and bio-medical-curative models of care approach or, instead, we can move towards integrated health systems that would be based on a socio-medical-preventive approach to health care.

## Background

Since the end of the last century, but especially at the beginning of this century, various health systems reform projects were initiated in the majority of middle income countries (MICs) which posed different degrees of complexity in their strategic proposals [1]. The main one has been the universal coverage strategy. Although this strategy has been defined from different perspectives, we basically return to the definition given by the World Health Organization (WHO): “Universal coverage refers to the strategy of guaranteeing universal access to comprehensive health services, preferably at a reasonable cost, without financial risks for the user, and avoiding falling into catastrophic health expenses [2].”

Since its creation, the Mexican health system was based on a mixture of the Bismarck and Beveridge models. Its structure and functioning are based on a legal framework that provides access to health services under different schemes for people with and without social security depending on their participation in the labor market and their status as salaried or not salaried. In the social security schemes, the main financing source is the federal budget via taxes; but employers and workers also contribute as a second source of financing. The most recent reform of the Mexican health system took universal coverage as its programmatic axis. It was proposed in 2002 and began its implementation in 2003 [3].

The complexity in the design and implementation of this strategy raises the need for some conceptual/operational clarifications to understand how to move from universal normative coverage to effective universal coverage. For the purposes of this analysis, we will consider the three stages that this strategy classically implies [4]. The first stage deals with universal affiliation, which refers to the adjustments that each country has to do in the legal framework in order to guarantee access to health services under public insurance financing. The second one is the development of a universal coverage scheme for health services, operated through a package of services that guarantees financial protection for all and according to health needs. The third stage refers to universal effective coverage, as a strategy implemented with equality to respond to the health needs of the population that is not protected by social security. This stage includes the implementation of a package of basic services that should be provided with good quality and that guarantees the reduction of health related out-of-pocket expenses or, in the best of cases, their elimination [5].

Most countries, mainly MICs, are trying to implement the three mentioned stages, which, although sequential, do require a gradual implementation or can be developed and implemented in parallel [6–8]. While it is true that major advances have been achieved in the legal framework and membership stage, the main challenges and dilemmas are found in stages 2 and 3, mainly in stage 3, which gives the evidence of efficiency and effectiveness of the scope of the reform on universal coverage. In this sense, taking evidence from the Mexican case [9, 10], the purpose of this manuscript is to focus on some evidence on what the third stage of implementation of universal coverage has achieved. It is interesting to analyze how we are moving from the discourse of universal coverage, in terms of legal framework, affiliation and model of care, towards an effective universal coverage that fully guarantees the greatest health benefits, covering the health needs of the population and avoiding the maximum possible out-of-pocket expenses for health reasons.

Once stages 1 and 2 have been reached, analyzing stage 3 implies highlighting and briefly defining the main barriers that are faced when transitioning to effective universal coverage. Indeed, to really achieve universal coverage as it is proposed in the new legal framework in health, the new affiliation schemes and the integral model of care, involves analyzing indicators of access, costs and utilization that guarantee the maximum effectiveness of such coverage strategies [11–13].

To analyze these indicators, we limit our object of study to the problems of diabetes and hypertension, which, together, represent the greatest magnitude and vulnerability. In addition, these two diseases are pressing the reform projects, particularly the coverage strategy. The comparison of normative universal coverage vs. effective coverage will be based on indicators of different interdependent variables. In fact, different components of the health system determine the behavior and balance between the health needs of the population and the complex response of the health system following the implementation of universal coverage strategies.

Summarizing, in a context of epidemiological changes and increasing financial requirements, and after 20 years of implementation of reform strategies in MICs, taking evidence from the Mexican case, the purpose of this manuscript is to highlight what has been achieved in universal coverage for the management of hypertension and diabetes. We are also interested in highlighting the main implications in terms of challenges, changes and dilemmas that need to be faced to achieve greater effectiveness in universal coverage goals.

## Methodological considerations

The focus of this essay was to identify the coverage trends for diabetes and hypertension and their implications since the implementation of the universal coverage strategy in a context of epidemiological and financial changes. For this purpose, the coverage strategy is addressed in two analytical categories [14]: normative universal coverage (crude coverage rate with no effective coverage and with some barriers depending on the demand for services for the control of hypertension) vs effective universal coverage (proportion of patients that effectively received care after demanding services to the health system for the control of hypertension).

The strategies addressing effective universal coverage that have been implemented consider all the determining factors presented above that are related to the interventions for diagnosis, treatment, adherence, and control that are being implemented in cases like Mexico. A package of basic services is included, with minimum quality standards, which should guarantee the effectiveness of interventions and the reduction of out-of-pocket expenses due to hypertension. In the best case, all the minimum inputs required for the good management and control of patients with hypertension (medicines, health personnel, infrastructure, supplies for promotion, detection, etc.) are guaranteed. The design and implementation are part of the national strategies and the progress achieved by crude coverage vs. effective coverage varies depending on the region or state concerned [14, 15].

We selected the sources of information considering the type of indicator [5, 9, 10, 16]. For epidemiological indicators, we used recent results of observed and expected epidemiological trends for diabetes and hypertension in Mexico at the national and state levels. To identify the coverage rates for both diseases, we used the official annual reports of the Health Secretariat of Health Performance for Chronic Diseases from 2005 to 2015. With regard to the financing indicators, we selected four studies that identified the economic burden for the health system and the users’ out-of-pocket expenses for diabetes and hypertension in 2005 and 2015 as key sources. The regions included in the analysis were those that, under different technical and feasibility criteria, had been selected in the studies we consulted on indicators for diabetes and hypertension in Mexico.

## Evidence of challenges facing health systems

**Fig. 1:**
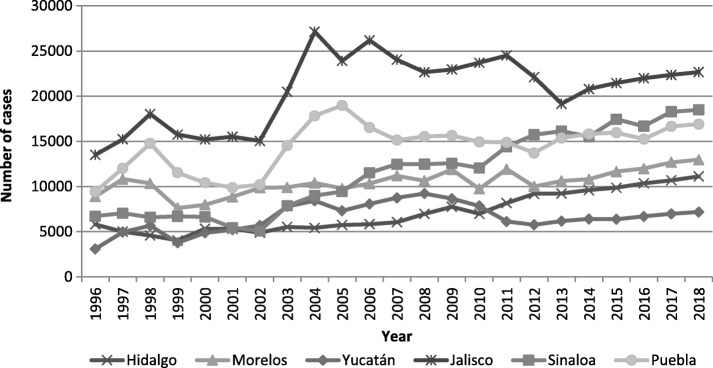
Trends of observed and expected cases for diabetes in selected states. Legend: This figure includes the annual data on the trend of cases of diabetes reported for 1998–2018 for six of the regions with the highest incidence and prevalence of diabetes in Mexico. Developed by authors with data from references [16, 17, 24, 31, 38]

**Fig. 2:**
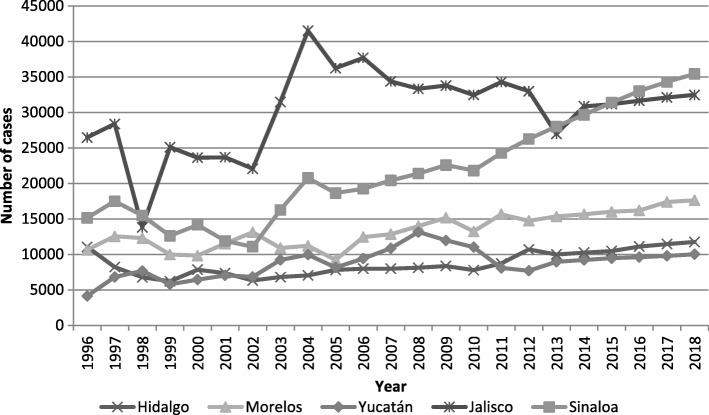
Trends of observed and expected cases for hypertension in selected states. Legend: This figure includes the annual data on the trend of cases of hypertension reported for 1998–2018 for six of the regions with the highest incidence and prevalence of hypertension in Mexico. Developed by authors with data from references [16, 17, 24, 31, 38]

**Fig. 3:**
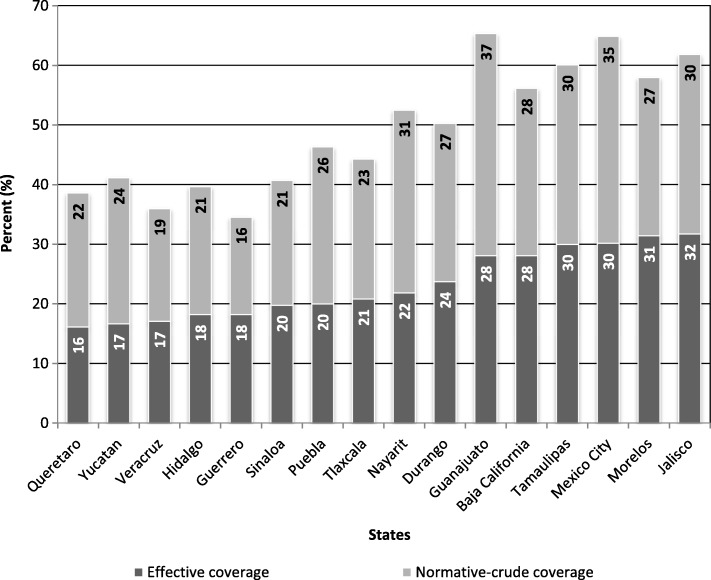
Results on normative-crude and effective coverage of hypertension treatment in selected states in Mexico, 2005–2015. Legend: This figure includes the trend of crude universal coverage rates and effective universal coverage for hypertension in the 16 regions of Mexico where there is greater progress in the implementation of universal coverage schemes in the context of health reforms. Developed by authors with data from references [5, 9, 10, 16, 38]

**Fig. 4:**
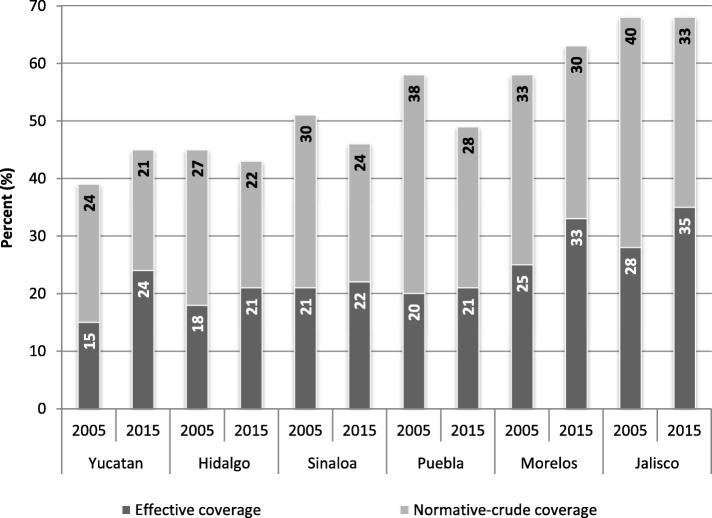
Effective coverage vs. normative-crude coverage for diabetes in selected states in Mexico, 2005–2015. Legend: This figure includes the trend of crude universal coverage rates and effective universal coverage for diabetes in the 16 regions of Mexico where there is greater progress in the implementation of universal coverage schemes in the context of health reforms. Developed by authors with data from references [5, 9, 10, 16, 38]

The contrast of normative universal coverage vs effective universal coverage is done in the form of lessons learned from Mexico, as an example of what is happening in the MICs, based on 4 categories of evidence:

- **Medium and short-term national indicators of epidemiological changes** in hypertension and diabetes (observed for 1995–2015 and expected for 2016–2018), obtained through the analysis of time series and autoregressive models. Figures 1 and 2 present the observed and expected trends for diabetes and hypertension cases from a time series analysis and forecast models under the Box-Jenkins technique [16, 17]. The observed cases concern the annual incidence of reported cases of hypertension and diabetes for adults over 20 years of age nationwide in the annual epidemiological report of the incidence of cases with medical diagnosis in one of the three main public health institutions [17]. The diagnosis was verified with blood glucose for patients with diabetes and with monitoring of blood pressure levels for patients with hypertension. In both cases, the international parameters for glucose and blood pressure levels were taken as reference [18–20]. This indicator highlights that, despite all the adjustments to health programs and health system reform strategies in place since the beginning of 2000, the observed and expected trends are constantly increasing.
- **Normative coverage indicators vs effective coverage** for the population over 20 years with hypertension at national level. In the case of normative coverage, indicators from the 2001 reforms, a set of interventions in response to health needs, were included in the metric. For effective coverage, estimates were derived for each state from records of effective use of health services for each disease [21, 22]. The results allow to identify gaps in the access to the intervention and places where the effectiveness of the intervention was suboptimal. Figure 3 shows the results for hypertension in a select group of states representative of the north, center and south of the country. For the national average, a minimum coverage of 50% was programmed from the public institutions of the national health system. The results showed a national average of 23% in effective coverage vs. 26% in raw coverage (programmed but not reached). The observed ranges for each type of coverage were 16–32% and 16–37% respectively. The summary of the data shows that, after 15 years of implementation of the coverage strategy, the national goal was met by more or less 50%.
- **Normative coverage indicators vs effective coverage** for population with diabetes at national level. We took two measurements, 2005 vs 2015, for the analysis of effective coverage [23, 24]. In this case, the data concern only the six states selected for the economic burden and epidemiology of diabetes in the Mexico study [16]. After 12 years of implementation of the coverage strategy, the results show an average of 60% coverage rates for these six states. The effective coverage range was 20–30% vs the normative coverage range of 30–35% (see Fig. 4).
- **Financial indicators of the requirements** to satisfy the real demand of health services for diabetes and hypertension. As a result of other studies [25–27], the observed and expected economic burden for both diseases was determined depending on the coverage rates. For this purpose, five indicators on financing were used: a) annual cost for effective coverage in 2015; b) estimated cost for normative-raw coverage in 2015; c) costs difference of effective coverage vs. normative-raw coverage; d) proportion of out-of-pocket expense in relation to total annual expenditure in 2005; e) percentage of out-of-pocket expense in relation to total annual expenditure in 2015. Regarding diabetes, the cost of effective coverage at the national level for 2015 was estimated at 4.5 billion dollars. While the expected raw coverage, if 100% of the concerned population had been attended to, was estimated at 10.45 billion dollars. Concerning hypertension, the cost at the national level was 3.2 billion dollars, vs. 7.4 billion dollars if 100% of the cases had been treated (see Table 1). In both cases, the cost of having covered 100% of the cases involved increasing all financial resources at least twice; resources of the health system as well as those from out-of-pocket payments made by the patients and their families.

**Table 1:** Some key indicators on the costs of effective coverage and normative-crude coverage for diabetes and hypertension in Mexico (in millions of dollars)

Indicator	Diabetes	Hypertension
Annual cost for effective coverage in 2015	4500	3200
Estimated cost for normative-raw coverage in 2015	5956	4200
Difference of costs effective coverage vs. normative-raw coverage	1456	1000
% of out-of-pocket expense in relation to total annual expenditure in 2005	49%	47%
% of out-of-pocket expense in relation to total annual expenditure in 2015	54%	50%

Developed by authors with data from references [16, 24–26, 38]

We also highlight the contribution of out-of-pocket expenses for both diseases comparing 2005 vs 2015. In both diseases, out-of-pocket expenses increase, although the increase is greater for diabetes, going from 49 to 53 dollars, out of every 100 dollars spent on diabetes in 2005 and 2015 respectively (see Table 1).

## Discussion

In most countries, the current organization and structure of health systems with public financing is one of the main barriers to guarantee effective universal coverage. In Mexico, as in most MICs, we have a fragmented system with three main subsystems: institutions for salaried workers of the state, institutions for salaried workers of private sector companies and institutions for non-salaried workers. It is in the latter that the greatest challenges of effective universal coverage are concentrated, above all, because of the high rates of catastrophic expenditure of its users [28, 29].

In systems as complex as health systems, we do not have conclusive evidence of improvement in coverage and financial protection at least for the 10 main health problems of the population [30]. The data presented here show an increase of out-of-pocket expenses for the two main causes of morbidity and mortality in Mexico. In the case of diabetes, the national average was 51 dollars out of every 100 dollars spent on health in 2005. It increased to 54 out of every 100 dollars spent on diabetes in 2015 [31]. In the case of hypertension, the results were very similar, 49 out of every 100 dollars of health expenditure in 2005 vs. 51 out of every 100 dollars of health expenditure coming from the patients’ pocket for 2015 [32]. These indicators show that the scope and achievements of effective universal coverage have not been sufficient to guarantee financial protection and reduce catastrophic spending, at least the one attributable to diabetes and hypertension.

Although in many countries there are no data to differentiate out-of-pocket spending by type of disease, the results are similar in countries such as India (70%), China (42%), Brazil (48%) and Russia (44%). We find a lower similarity when comparing Mexico with higher-income countries such as Luxembourg (16%), France (19%), Turkey (21%) and Spain (26%) [33].

On the other hand, when compared with other countries, according to the first global monitoring report on universal coverage in 2015 [34], the results for Mexico are within the range reported for all the analyzed countries; although, Mexico has better results in the case of effective universal coverage for hypertension. The range observed in 96 selected countries for normative / crude coverage in hypertension was 7–61% and for effective coverage 11–31%; highlighting Peru, Qatar, Egypt, Kyrgyzstan and Cambodia as the countries with the highest rates of effective coverage. While the coverage rate for diabetes was in the range of 3–70% in normative/crude coverage (without totally covering treatment and control) and 4–60% in effective coverage (covering diagnosis, treatment and medicated control), highlighting countries such as Qatar, Moldova, Lao and Uzbekistan.

Concerning the analysis of the economic sustainability that guarantees financial protection against health damages, the health systems of MICs do not have guidelines, and validation of strategic planning models would allow identifying and managing financial imbalances between supply and demand in health, when analyzing the scope of universal coverage [35–37]. From the perspective of complex systems, it is necessary to identify needs and financing requirements in health for the challenges of chronic diseases within the framework of effective universal coverage, starting from real situations vs. objective image. In this sense, the generation of new financing alternatives and the conformation of resource allocation patterns are two of the planning strategies that health systems have not yet been able to solve to guarantee effective coverage.

The first reform programs proposed to implement strategic changes to generate more and new economic resources, in addition to making a more effective planning. However, in the totality of the public health sub-systems in Mexico, dependence on the same financing sources that operated since the 1970s persists [38]. Besides, there has been no progress in the allocation of resources, which is usually based on the annual historical trends by type of institution, as at the central government levels [39].

The analysis and discussion need to include complexity principles to change the debate on the “behavior change” of the health system and the identification of all its determinants. In fact, from the perspective of complex systems, important changes are needed in terms of health policies and hospital administration in order to face the challenges of universal normative coverage vs. effective universal coverage.

The evidence presented was generated from a systematic analysis that involved an integral and transdisciplinary perspective including disciplines such as anthropology, economics, epidemiology, clinical, public health, administration and political science. From this perspective, we consider that, in order to assure the effectiveness of the universal coverage strategy, fundamental changes are required in the organizational structure and in the model of care. Particularly in the model that was launched in the 1940s and is still functioning in 2018 in Mexico and in most MICs.

We must also recognize that, at the global level, health systems have not been able to solve the population’s demands for care, mainly in relation to non-communicable diseases. Moreover, not only we have not been able to meet the health needs for diabetes and hypertension with effective coverage, but such demands will increase exponentially. This means that, in the coming decades, trying to reach effective coverage could lead to financial collapse of health systems with more serious repercussions for the patient’s pockets.

After 20 years of reforms and 15 years of having implemented the new universal coverage schemes, and in spite of important progress, results are not yet satisfactory. The effective coverage reached for the management and control of hypertension and diabetes does not go beyond 50% of compliance. The goal to cover 100% of the demand for services related to these two diseases will continue pending. It will be difficult to reach a greater or total effective coverage if there are no adjustments and fundamental changes, both in the strategy per se and in the model of care. In this sense, the greatest implication of our analysis is that there are four categories of challenges facing the universal coverage strategy:

- **The challenges of the epidemiological transition.** Since the end of the last century in most MICs, the needs and health conditions of the population have moved from an epidemiological model based on communicable diseases to a mixed epidemiological model mainly based on non-communicable diseases and accidents [40], but with the permanence of certain transmissible diseases. This contemporary or dilated model of the transition describes an incomplete and mixed transition phenomenon of most MICs. Despite the existing changes, the decrease in mortality was delayed until the third, fourth or fifth decade of the last century [41]. In most of the countries where this model is present, mortality dropped dramatically after the end of World War II, while fertility was maintained at high levels [42]. The challenges posed by this transition phenomenon are related to the lack of balance between supply and demand, as well as to the financial imbalances generated by the changing demand for health services [43, 44].
- **The challenges of the health system and model of care.** In spite of several generations of health reforms implemented in the last 20 years, in Mexico as in the majority of MICs, we have a health system that originated in the 1940’s and that is still in place as of 2018 with the original organic and functional structure [45, 46]. In addition to a policy of efficiency in the search and allocation of financial resources, it is also necessary to develop policies to strengthen financing of promotion, prevention and control of health damages, mainly for non-communicable diseases such as diabetes and hypertension. In this sense, the main health policy we must discuss with strategic lines is to shift from a model of care based on a bio-medical, curative and fragmented approach, towards a model of care based on a socio-medical, comprehensive and preventive focus.
- **The challenges in the training of health professionals.** The health professionals training model is still based on the Flexner model, a model designed and implemented in 1910 to address the health problems of North American society and subsequently exported and adapted to the rest of the Western world [47]. Certainly, this is the origin and greatest ally of the current biomedical-healing model in all MICs. The health professionals training model requires substantive adjustments in its content and form. In terms of form, health professionals must develop more skills to focus and resolve health damage from a perspective of promotion and prevention and the social determinants of health. In terms of content, promotion and prevention skills should be focused on the list of the main diseases that characterize each region or country at a population level. The medicine of this century should be equally nourished by biological sciences as social sciences [48]. Moreover, as proposed by the WHO, we dare to state that the social sciences could fill the gaps that medicine, biological and health sciences have not been able to solve [49].
- **The challenges of the economic burden.** In economic terms, the cost-effectiveness of the state of the art of medicine has not been as expected. State of the art evidence based medicine [50–53] has shown efficacy and effectiveness in solving health problems such as diabetes and hypertension. However, at a population level, the problem is not only unresolved but keeps growing and pressing everyone: patients/families, health systems and society as a whole. The direct costs of care for patients and for the health system grow exponentially. Indirect costs due to temporary and permanent disability, as well as premature death and avoidable deaths, grow at an even higher rate for society. The economic gain of this transition in the care model would generate high benefits in reducing the economic burden of damages attributable to diabetes and hypertension. Evidence from simulation models shows that increasing the resources in promotion and prevention in 30% could produce potential savings of 70–80% of the current economic burden for diabetes and hypertension [54, 55].

Given the challenges posed, the dilemma between continuing with the same model of care and coverage programs and proposing a rupture cannot be postponed. As we pointed out at the beginning of this manuscript, health systems have not guaranteed greater effectiveness according to the state of the art medicine, nor greater effectiveness in the resolution of the management and control of cases through effective coverage. We must then, at least, discuss and propose a gradual transformation of the model of care that has operated in the last 20 years and that has not been able to meet the expected demand for diabetes and hypertension.

A limitation of our analysis, in the first place, is that using indicators of secondary databases does not allow going beyond the results of the indicator itself. The measurement of effective coverage vs normative-crude coverage does not allow analyzing the determinants of low to high levels of effective coverage depending on the region of the country or the management of the disease in detail. The coverage scheme itself does not allow to identify the effects of the epidemiological changes for each disease in terms of the changes required based on the demand for new cases diagnosed and changes required in the reallocation of financial resources. Another important limitation, both in our analysis and in the use of the indicator, is that the universal coverage strategy and its monitoring may disappear. Indeed, there is uncertainty about the continuity of the health reform project and the universal coverage strategy. In Mexico, a substantive change is highly probable in the new National Health Program 2019–2025, which will begin in 2019 with a new public administration at the federal level.

## Conclusion

Undoubtedly, not having health systems that can guarantee effective universal coverage implies a critical moment for state of the art medicine. The evidence presented for the Mexican case using diabetes and hypertension as tracers show the dilemma and the main challenges that health systems, patients, families and society as a whole, are and will be facing in the following years in Mexico and, probably, in most MICs. After 20 years of general health system reforms and 15 years of social protection strategies and universal coverage, MICs’ health systems have not been able to guarantee effective universal coverage. Moreover, financial protection for patients and the goal of reducing catastrophic expenses for health reasons have not improved, they have even worsened. This is what we can see in the evidence regarding hypertension and diabetes.

Faced with the dilemma of continuity or rupture, we bet on a gradual rupture. We invite all international and national agencies and actors involved in health reforms in general and in the universal coverage strategy in particular to review, analyze, discuss and develop strategic lines that allow a behavioral change from all domains, resources and actions of complex health systems. The model of care based on a curative biomedical approach sufficed to respond to the health needs of the last century, but not for the needs of the present century. We need to move from models based on a bio-medical, curative and fragmented approach to a new model of care based on a socio-medical, preventive and comprehensive approach. Only in this way can we advance in the goals of effective coverage responding to the main health needs of the population. Only then, the contributions of state of the art medicine will be effective in the resolution of each specific health problem in the complexity that transits from universal normative coverage to effective universal coverage.
